# Product Review of SR Accelerator Deduplicator

**DOI:** 10.29173/jchla29737

**Published:** 2024-04-01

**Authors:** Rachel Couban

**Affiliations:** Research Coordinator, McMaster University, Hamilton, ON, Canada

**Product:** SR-Accelerator Deduplicator

**URL:** https://sr-accelerator.com/#/deduplicator

## Purpose

To remove duplicate citations from systematic review searches. Typically, we search multiple databases for systematic reviews. The content coverage of the various databases overlaps to varying degrees, and duplicate citations are retrieved. It is a painstaking process to remove the duplicates from a set of search results. SR-Accelerator helps to process the duplicate citations.

## Product/resource description

The SR-Accelerator Deduplicator tool is easy to use and does a beautiful job removing duplicate citations from sets of articles. You simply upload the full set, use the tool to process the duplicates, and download your deduplicated results. At the processing level, you are guided through four sets of potential duplicates, organized by how quickly or thoroughly you should check each set.

## Intended audience/users

Librarians, library staff, students, or anyone supporting a systematic review team to search multiple databases, download the results and remove duplicates.

## Special features

A great feature of the SR-Accelerator is the option to Remove Already Screened & Deduplicate, which is excellent for systematic review search updates. Simply load last year’s batch into the Already Screened Articles box, and drop the new results into the New Articles to Deduplicate box, and you can check your new search results against the set that were already screened.

## Compatibility issues

I’m used to using the SR-Accelerator with EndNote, but any bibliographic software could be used as long as one of the main formats is used (XML, RIS, NBIB.) I used to use the XML format, but recently I have started using the RIS format and it works wonderfully.

## Platform

The SR-Accelerator is a web-based platform, available at https://sr-accelerator.com/#/deduplicator.

## Usability

There is a help page that gives step-by-step instructions, and there are lots of steps! Although the tool is easy to use, processing the duplicates is not effortless. For large sets the task of removing duplicates is still painstaking. But with this tool you feel that the computer is doing most of the hard work for you.

## Strengths and weaknesses

Since I started using this software during the pandemic, it has always worked beautifully with no problems. It adds another step to the workflow because you upload the set, process the duplicates, and download them, all of which introduce the chance of errors.

## Comparison with similar products

Before this I used the EndNote Deduplication method described by Wichor Bramer [1] Using Bramer's method, you change the default deduplication settings to use combinations of various fields that define duplicates. It is effective but feels tedious once you have used the Deduplicator. I have also seen people posting to a library listserv about a new tool called Deduklick which removes duplicates [2]. But I have not explored that product.

## Currency

N/A

## Cost/value

Free

## Contact information

theteam@sr-accelerator.com

Bond University Institute for Evidence Based Healthcare.
